# Minor Surgery and Bandaging

**Published:** 1891-11

**Authors:** 


MINOR SURGERY AND BANDAGING, including the Treatment of Fractures and Dislocations, Tracheotomy, Intubation of the Larynx, Ligations of Arteries, and Amputations. By HENRY R. WHARTON, M. D., Demonstrator of Surgery and Lecturer on Surgical Diseases of Children in the University of Pennsylvania, etc. In one very handsome 12mo volume of 491 pages, with 403 illustrations. Enameled cloth, $3.00. Philadelphia: Lea Brothers & Co. 1891.

The surgery of the present period, which may be properly called the new surgery,—the surgery of antisepsis, of simplicity, and of cleanliness,—is also a surgery of success and cure ; but the very conditions which have advanced surgery to its present state of triumph, have also necessitated the re-writing of most of the treatises that were formerly accepted as guides, or else of the publication of entirely new works cast upon the modern lines of pathology and treatment. This is as clearly true of the subject of bandaging as of any subordinate branch on the department of general surgery. Dr. Wharton has furnished an admirable treatise on Minor Surgery and Bandaging, which also includes many of the lesser details that are not to be found in the more elaborate treatises that are called text-books. In our opinion, the present work should be adopted as a text-book in our colleges, for it contains more practical surgery within its limits and boundaries than any book of its kind we have ever seen. The illustrations are especially good, made from clear and correct drawings, or else have been reproduced from photographs. It is difficult to see how even a tyro could fail to understand the application, for instance, of the dressing for fracture of the upper humerus, after looking at the picture on page 322. Some of the cuts in this book are recognized as old friends, taken from Liston, Cooper, and Fergusson, but such are generally classic illustrations of given conditions, upon which no improvements could be made. One of the strongest sections of the book is that upon Sutures, which we commend for the clearness of the illustrations, as well as the text. Taken altogether, we are more than satisfied with this attempt to place before the profession an abridgement of general surgery, which shall be instructive and scientific, without, at the same time, being classed among those books that are prepared for the purpose of cramming a student for final examination. Minor surgery has become a legitimate subdivision of the general field, and such being the case, it is well to have the best literature possible. In the present case, no mistake has been made in placing the preparation of such a treatise in the hands of Wharton, who has proven himself up to his work.

The publishers have put the volume into an attractive and substantial dress.

